# Supplementary material for: Gas Phase Emissions of Volatile Organic Compounds Arising from the Application of Sunscreens
Source: Int J Environ Res Public Health. 2023 May 24;20(11):5944. doi: 10.3390/ijerph20115944 (PMC10252547; doi:10.3390/ijerph20115944)

Supplementary Information

Table S1: Classification of the 18 randomly selected PCPs for petroleum/organic ethanol for VOC contaminant analysis.

Petroleum Derived Ethanol PCPs		Organic Ethanol PCPs	
Body Wash	P1	Moisturiser	O1
Face Wash	P2	Liquid Hand Soap	O2
Conditioner	P3	Conditioner	O3
Foundation	P4	Face Wash	O4
Foundation	P5	Moisturiser	O5
Moisturiser	P6	Moisturiser	O6
Moisturiser	P7	Bubble Bath	O7
Moisturiser	P8	Body Lotion	O8
Foundation	P9	Body Lotion	O9

Table S2: SIFT-MS SIM method targeted scanned m/z values, and their corresponding ions, for each of the three reagent ions $H3O^+$, NO^+, and $O2^+$

Compounds	H_3O^+ *m/z*	NO^+ *m/z*	O_2^+ *m/z*
2-Propanol		59 ($C_3H_7O^+$)	45 ($C_2H_5O^+$)
Benzene		78 ($C_6H_6^+$)	78 ($C_6H_6^+$)
Benzyl Acetate		150 ($C_9H_{10}O_2^+$)	
Benzyl Alcohol		107 ($C_7H_7O^+$)	107 ($C_7H_7O^+$)
D5*	355 ($C_9H_{27}O_5Si_5^+$)<br>371 ($C_{10}H_{31}O_5Si_5^+$)	355 ($C_9H_{27}O_5Si_5^+$)	355 ($C_9H_{27}O_5Si_5^+$)
Di1**	237 ($C_8H_{25}O_2Si_3^+$)<br>239 ($C_7H_{23}O_3Si_3^+$)	239 ($C_7H_{23}O_3Si_3^+$)	239 ($C_7H_{23}O_3Si_3^+$)
Di2***	295 ($C_9H_{27}O_3Si_4^+$)<br>313<br>($C_9H_{27}O_3Si_4.H_2O^+$)	295 ($C_9H_{27}O_3Si_4^+$)<br>313<br>($C_9H_{27}O_3Si_4.H_2O^+$)	295 ($C_9H_{27}O_3Si_4^+$)<br>313<br>($C_9H_{27}O_3Si_4.H_2O^+$)
Ethanol	47 ($C_2H_7O^+$)<br>65 ($C_2H_7O.H_2O^+$)	45 ($C_2H_5O^+$)<br>63 ($C_2H_5O.H_2O^+$)	
Ethyl Acetate	89 ($C_4H_9O_2^+$)	118 ($C_4H_8O_2.NO^+$)	88 ($C_4H_8O_2^+$)
Ethylbenzene		106 ($C_8H_{10}^+$)	106 ($C_8H_{10}^+$)
Hexyl Acetate		174 ($C_8H_{16}O_2.NO^+$)	
Limonene****	137 ($C_{10}H_{17}^+$)<br>155 ($C_{10}H_{17}.H_2O^+$)	136 ($C_{10}H_{16}^+$)	136 ($C_{10}H_{16}^+$)<br>137 ($C_{10}H_{17}^+$)
t-Butyl Alcohol	57 ($C_4H_9^+$)	57 ($C_4H_9^+$)	59 ($C_3H_7O^+$)
Toluene		92 ($C_7H_8^+$)	
Trimethylsilanol		93 ($C_2H_9O_2Si.H_2O^+$)	

*Decamethylcyclopentasiloxane

**Octamethyltrisiloxane

***Decamethyltetrasiloxane

****Representing monoterpenes grouped

Table S3: Emission Factors of VOCs from 26 different sunscreens for 15 of the most commonly found and abundant species. Excluding from the statistics are products where QTOF results showed a particular VOC was not present in the original formulation

Product	Emission Factor (µg s^{-1} $g_{[Product]}^{-1}$)														
	2-Propanol	Benzene	Benzyl Acetate	Benzyl Alcohol	D5	Di 1	Di 2	Ethanol	Ethyl Acetate	Ethylbenzene	Hexyl Acetate	Limonene	t-Butyl Alcohol	Toluene	TMS
S1	4.07E-02	3.78E-04	5.05E-02	6.87E-03	5.71E-04	2.93E-03		7.94E+00	1.46E-03		1.24E-03	1.51E-03	4.55E-02	1.02E-02	1.79E-01
S2	1.23E-02					1.32E-03	2.04E-03	3.30E+00	1.37E-03	7.56E-04	2.43E-04	1.50E-03	2.83E-02		8.34E-03
S3	5.06E-03		1.04E-03		3.28E-04	3.25E-03	6.17E-03	7.92E-01	3.62E-04	4.42E-04	2.51E-04	9.29E-04	2.16E-02	8.67E-05	3.46E-03
S4	3.78E-02	3.68E-03	1.07E-02	4.18E-03	1.79E-04	1.65E-03	8.65E-04	1.51E-02	5.21E-04	1.58E-02	7.40E-04	1.70E-02	5.78E-02	8.01E-02	7.46E-03
S5	1.20E-02	1.04E-02	2.08E-02	1.71E+00	3.12E-01	8.43E-04	1.47E-03	7.38E-03	4.47E-04	2.29E-03			6.36E-02	2.93E-02	2.00E-03
S6	6.05E-01	6.55E-04	2.40E-03			4.56E-03	2.33E-03	3.06E+00	1.39E-03	1.91E-04		1.75E-03	1.21E-01	4.96E-03	4.56E-02
S7	4.89E-02		1.22E-01	3.90E-01	4.97E-04	3.78E-02	8.02E-02	5.60E-02	2.54E-02	9.15E-03	9.91E-03	1.47E-02	3.84E-01	1.38E-02	4.19E-02
S8	5.50E-03	3.65E-03	4.00E-03	6.80E-02	2.07E-03	4.56E-02	1.06E+02	3.38E-02			7.03E-03	5.61E-03	1.43E-01	5.89E-04	2.35E-02
S9	8.42E-01			3.22E-02	8.11E-04	2.24E-03	2.77E-02	4.48E+00	1.47E-03		9.16E-04	1.86E-03	1.33E-02	3.78E-03	7.35E-02
S10	1.30E-02	7.27E-04	5.69E-02	4.50E-02	2.44E-04	1.30E-03	8.17E-03	1.05E-02	3.30E-04	2.31E-03	2.67E-02	5.10E-02	1.33E-02	6.04E-03	6.00E-03
S11	1.19E-02				1.24E-04	3.95E-03		1.65E+00	1.37E-03		3.75E-02		4.81E-02	3.54E-02	
S12	3.01E+00	1.88E-03	2.35E-02	4.85E-02	2.94E-06	2.56E-03		1.08E+01	4.49E-03	1.22E-03	2.73E-04		2.44E-01	5.26E-02	2.38E-01
S13	3.22E-03	8.21E-04	1.27E-01	3.53E-02		8.73E-04	2.00E-02	1.66E-02	2.24E-04	3.84E-03	7.91E-04	3.35E-02	1.71E-02	1.72E-02	1.78E-02
S14	6.28E-02	4.40E-04	9.06E-03	3.55E-03	3.72E-04	8.39E-02	2.08E-01	8.40E-03	4.01E-04	1.44E-03			1.81E-01	1.58E-03	5.91E-03
S15	7.17E-03	4.41E-04	3.85E-03	1.15E-04				1.55E+00	3.80E-04	2.81E-03	1.02E-03		4.65E-02		
S16	8.23E-03	9.13E-04	3.84E-02	2.83E-05	4.29E-04	2.69E-03	1.10E-02	6.60E-04	6.15E-04	3.40E-03			7.96E-02		4.61E-03
S17	4.67E-03	3.01E-05	1.13E-02	2.23E-03			1.96E-02	6.83E-03	5.39E-04		1.87E-03	9.63E-03	3.36E-02	1.74E-03	2.08E-03
S18	9.19E-03	2.15E-03	7.76E-03	4.18E-03		2.36E-03	1.82E-02	2.14E-02	8.54E-04	2.86E-03	6.19E-04		4.78E-02	1.92E-02	9.95E-03
S19	2.46E-02	2.48E-03	2.56E-02	2.97E-01	6.68E-02		1.44E+02	5.27E-02	1.34E-02	6.45E-03	2.85E-03	2.53E-03	2.54E-01	1.15E-03	1.33E-02
S20	4.98E-02	8.27E-05	5.98E-02	1.52E-01				5.98E+00	1.90E-03	6.89E-03	1.48E-03	6.00E-03	2.80E-01	1.31E-02	9.89E-02
S21	1.16E-02	4.32E-04	1.73E-03	1.19E-02	3.12E-04		1.04E-01	2.99E+00	8.75E-04	8.04E-04	4.28E-02	2.44E-03	1.20E-01	3.33E-03	8.86E-02
S22	9.43E-03	9.08E-04	3.33E-02	1.28E-01	4.40E-04		2.96E-02	2.58E-01	5.80E-04	4.74E-03	5.27E-02	1.48E-01	6.02E-02	1.63E-02	1.52E-02
S23	2.54E-02	7.13E-04	2.59E-02	5.56E-02	2.88E-04	5.31E-04	3.20E-02	8.20E-03	3.94E-05	2.29E-04			1.32E-01	2.98E-04	
S24	2.09E-01	4.57E-03	3.64E-02	6.10E-02			8.06E-03	1.58E+00	2.93E-04	1.56E-03	2.03E-03	1.20E-01	2.23E-01	7.87E-02	4.33E-02
S25	9.26E-03		4.35E-02	3.07E-02		1.14E-03		3.34E-01	2.53E-04	2.69E-03	7.12E-04	1.78E-02	7.73E-02		1.00E-02
S26	3.13E-03	1.43E-03	7.66E-02	2.57E-02	8.34E-05	3.49E-03	3.26E-02	1.66E-02	4.02E-04	4.84E-04	8.90E-04	3.54E-02	8.53E-02	3.68E-02	1.76E-02
Mean	1.95E-01	1.84E-03	3.44E-02	1.41E-01	2.27E-02	1.07E-02	1.25E+01	1.73E+00	2.37E-03	3.35E-03	9.17E-03	2.61E-02	1.09E-01	1.94E-02	4.16E-02

Table S4: Inhalation estimates for individual VOCs based on a single facial application of sunscreen presented in Table 4 and calculated headspace/inhaled ratios.

Species	Estimated inhalation for head, neck, and face application based on age and sex (mg)						
	Men (16+ years)	Women (16+ years)	Child (13-15 years)	Child (11-12 years)	Child (8-10 years)	Child (5-7 years)	Child (2-4 years)
Benzene	7.86E-03	6.92E-03	6.29E-03	5.47E-03	4.41E-03	3.43E-03	2.58E-03
Benzyl Acetate	2.65E-01	2.34E-01	2.12E-01	1.85E-01	1.49E-01	1.16E-01	8.72E-02
Benzyl Alcohol	4.08E+00	3.59E+00	3.27E+00	2.84E+00	2.29E+00	1.78E+00	1.34E+00
D5	2.96E-01	2.61E-01	2.37E-01	2.06E-01	1.66E-01	1.29E-01	9.72E-02
Dimethicone 1	9.40E-02	8.28E-02	7.53E-02	6.54E-02	5.27E-02	4.10E-02	3.09E-02
Dimethicone 2	1.26E+02	1.11E+02	1.01E+02	8.78E+01	7.07E+01	5.51E+01	4.15E+01
Ethanol	2.97E+01	2.62E+01	2.38E+01	2.07E+01	1.66E+01	1.30E+01	9.76E+00
Ethyl Acetate	5.98E-03	5.27E-03	4.79E-03	4.16E-03	3.35E-03	2.61E-03	1.97E-03
Ethylbenzene	2.34E-02	2.06E-02	1.88E-02	1.63E-02	1.31E-02	1.02E-02	7.70E-03
Hexyl Acetate	1.84E-01	1.62E-01	1.48E-01	1.28E-01	1.03E-01	8.04E-02	6.05E-02
Limonene	2.38E-01	2.10E-01	1.91E-01	1.66E-01	1.33E-01	1.04E-01	7.82E-02
2-Propanol	4.43E-01	3.90E-01	3.55E-01	3.08E-01	2.48E-01	1.93E-01	1.46E-01
t-Butyl Alcohol	1.96E-01	1.73E-01	1.57E-01	1.37E-01	1.10E-01	8.57E-02	6.46E-02
Toluene	1.54E-01	1.36E-01	1.24E-01	1.07E-01	8.65E-02	6.74E-02	5.07E-02
Trimethylsilanol	6.34E-02	5.59E-02	5.08E-02	4.42E-02	3.56E-02	2.77E-02	2.09E-02
Total	1.62E+02	1.43E+02	1.30E+02	1.13E+02	9.08E+01	7.07E+01	5.32+01

Table S5: Gas phase mixing ratios of contaminant species off-gassed from sunscreens for each of the 26 sunscreen products.

Product	Benzene (ppb)	Toluene (ppb)	Ethylbenzene (ppb)
S1	0.3	6.2	
S2			
S3			0.6
S4	4.7	86.7	14.8
S5		37.2	
S6		3	
S7			
S8			
S9			
S10			
S11		8.3	
S12		26.5	
S13	0.5		1.6
S14		1.8	
S15			1.3
S16			2.2
S17			
S18	1.5		1.5
S19			3.5
S20			
S21		2.7	
S22			
S23			
S24		56	
S25			
S26	1.2	26.6	
Mean	1.6	25.5	3.6

Figure S1: Liquid calibration curves for four compounds. Points represent SIFT-MS measured mixing ratio. Solid line represents linear regression for SIFT-MS measured mixing ratios. Dashed lines represent delivered mixing ratio.

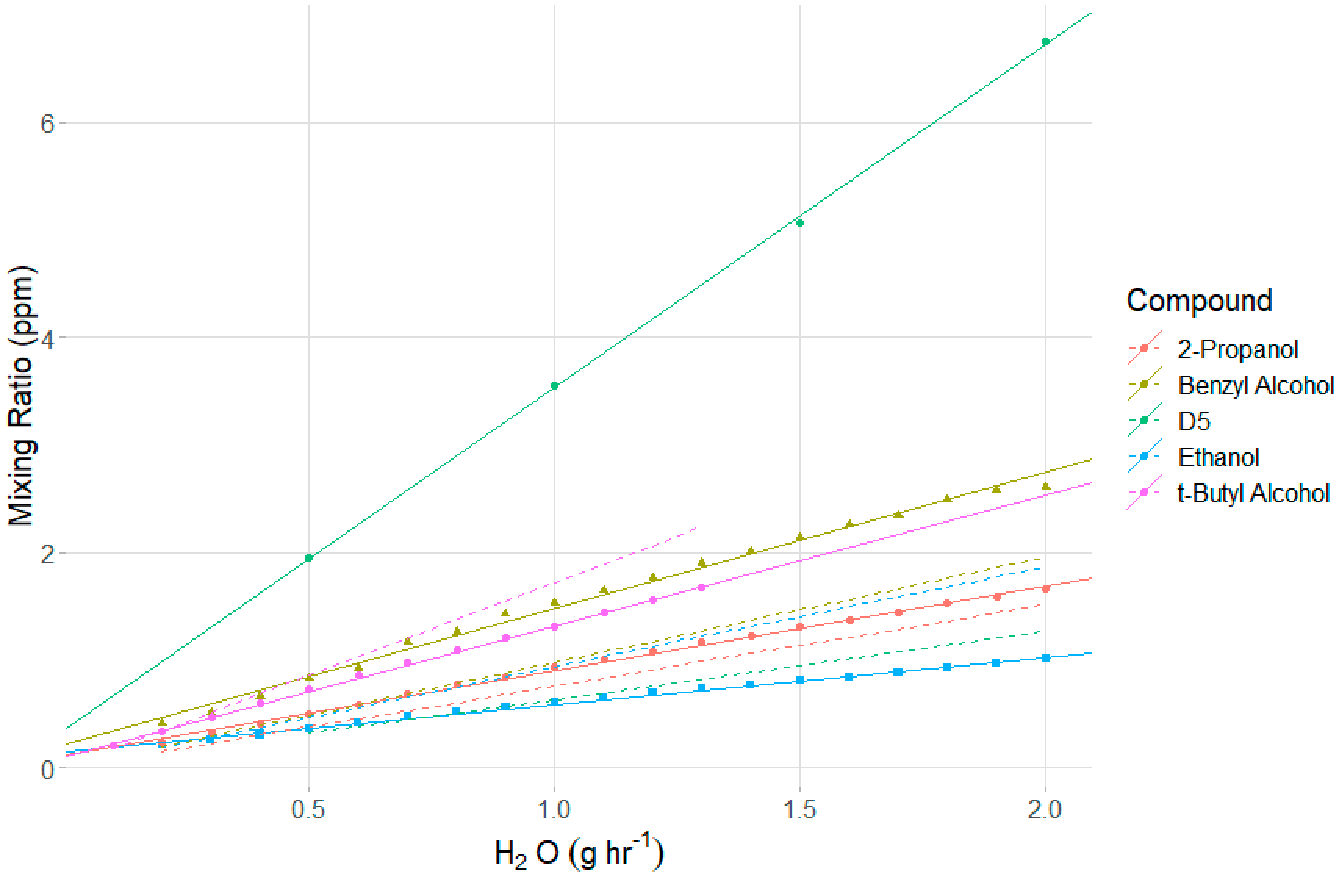

Supplement: Supplementary file 1 [file ijerph-20-05944-s001.zip › ijerph-2298988-supplementary.pdf]
